# Good cop, bad cop: challenging patients to make hard decisions about aggressive treatment

**DOI:** 10.1186/1757-1146-4-S1-P50

**Published:** 2011-05-20

**Authors:** Katrina Richards

**Affiliations:** 1 Podiatry Department, Box Hill Hospital, Eastern Health, Australia

## 

This is a case study that focuses on two acute presentations of diabetic foot ulcerations. Both men had a history of previous ulceration and presented to the Emergency Department at Box Hill Hospital with acutely infected weight-bearing foot ulceration caused by blistering. Both men worked in manual occupations and were subsequently weight-bearing for a large part of the day. In both cases a Total Contact Cast was decided to be the most effective means of treatment. Upon discharge from hospital, one of the men took a week’s sick leave from him occupation and his ulceration improved rapidly, so much so that he didn’t require a second week of total contact casting. The wound quickly healed and he has had no further problems to date and has returned to work as usual. The second man was in a Total Contact Cast for one week. On review, the Podiatrist determined that he would benefit from a second week in a TCC. The patient refused this treatment as it was inconvenient to his workplace and he didn’t like the TCC. He was treated with a padded CAM walker. Unfortunately he presented with an acute infection one week later and subsequently required a transmetatarsal amputation. He also had to take several weeks sick leave from his workplace and had to be slowly rehabilitated into the workplace post-amputation. These cases have highlighted the benefits of aggressive treatment when dealing with weight-bearing diabetic foot ulcerations. Quite often as Podiatrists, we don’t want to cause inconvenience to the patient’s working life, but this may in fact be to the patient’s detriment. Sometimes to provide the best patient centred care, we need to be the ‘bad cop’ and make unpopular decisions that will offer the best results.

